# Dr. Patterson's Case of Angine Pectoris

**Published:** 1803-07-01

**Authors:** 


### *To the Editors of the Medical and Physical Journal.*

GENTLEMEN,

I Beg leave to state to you a few Observations that have occurred to me, upon a subject of great importance to the Medical Faculty at this momentous period.

For the necessary defence of these kingdoms in the present situation of public affairs, it seems that a new Income Tax is to be adopted, and an additional military force is to be raised. I am happy on many accounts to see, that a large sum of money is to be produced within the year, instead of resorting to the funding system for our supplies; but unless medical receipts be calculated with great tenderness, the contributions from them will be proportionably greater than from the other classes in society. The stability of medical practice is peculiarly uncertain, and the establishments of the Faculty are necessarily considerable; consequently, it seems extremely hard to rate their incomes equally with such as are more durable, and do not

---

not require servants, horses, or carriages for their production. Again, in the proposed plan for national defence, I think it will be reasonable to excuse the military services of every medical practitioner who agrees to contribute his professional assistance whenever the enemy lands upon the coasts of these kingdoms, or when the country is in *imminent danger* of being invaded. I will not enlarge upon either of these topics, because I presume that the propriety and force of them must be acknowledged by every one; and I think, if they were properly represented to Government, some relief would be afforded to us. I therefore recommend that a meeting of Physicians, Surgeons, and Apothecaries should be immediately called, and some plan of harmony and co-operation entered into, in order to place the Faculty upon an equal footing with the other classes of society. I am, &c.

*June* 20, 1803. MEDICUS.

